# LRRK2 levels in immune cells are increased in Parkinson’s disease

**DOI:** 10.1038/s41531-017-0010-8

**Published:** 2017-03-28

**Authors:** D. A. Cook, G. T. Kannarkat, A. F. Cintron, Laura M. Butkovich, Kyle B. Fraser, J. Chang, N. Grigoryan, S. A. Factor, Andrew B. West, J. M. Boss, M. G. Tansey

**Affiliations:** 1 Department of Physiology, Emory University School of Medicine, Atlanta, GA USA; 2 Department of Neurology, Center for Neurodegeneration and Experimental Therapeutics, University of Alabama at Birmingham, Birmingham, AL USA; 3 Department of Neurology and Movement Disorders Center, Emory University School of Medicine, Atlanta, GA USA; 4 Department of Microbiology and Immunology, Emory University School of Medicine, Atlanta, GA USA

## Abstract

Mutations associated with leucine-rich repeat kinase 2 are the most common known cause of Parkinson’s disease. The known expression of leucine-rich repeat kinase 2 in immune cells and its negative regulatory function of nuclear factor of activated T cells implicates leucine-rich repeat kinase 2 in the development of the inflammatory environment characteristic of Parkinson’s disease. The aim of this study was to determine the expression pattern of leucine-rich repeat kinase 2 in immune cell subsets and correlate it with the immunophenotype of cells from Parkinson’s disease and healthy subjects. For immunophenotyping, blood cells from 40 Parkinson’s disease patients and 32 age and environment matched-healthy control subjects were analyzed by flow cytometry. Multiplexed immunoassays were used to measure cytokine output of stimulated cells. Leucine-rich repeat kinase 2 expression was increased in B cells (*p* = 0.0095), T cells (*p* = 0.029), and CD16^+^ monocytes (*p* = 0.01) of Parkinson’s disease patients compared to healthy controls. Leucine-rich repeat kinase 2 induction was also increased in monocytes and dividing T cells in Parkinson’s disease patients compared to healthy controls. In addition, Parkinson’s disease patient monocytes secreted more inflammatory cytokines compared to healthy control, and cytokine expression positively correlated with leucine-rich repeat kinase 2 expression in T cells from Parkinson’s disease but not healthy controls. Finally, the regulatory surface protein that limits T-cell activation signals, CTLA-4 (cytotoxic T-lymphocyte-associated protein 4), was decreased in Parkinson’s disease compared to HC in T cells (*p* = 0.029). In sum, these findings suggest that leucine-rich repeat kinase 2 has a regulatory role in immune cells and Parkinson’s disease. Functionally, the positive correlations between leucine-rich repeat kinase 2 expression levels in T-cell subsets, cytokine expression and secretion, and T-cell activation states suggest that targeting leucine-rich repeat kinase 2 with therapeutic interventions could have direct effects on immune cell function.

## Immune system regulation: A known suspect

High levels of leucine-rich repeat kinase 2 (LRRK2) in immune cells disrupt immune system function in patients with Parkinson’s disease (PD). Mutations in LRRK2 are the most common genetic cause of PD. Although LRRK2 is found in many immune cells, research efforts have focussed on determining its effects on neuronal function. Malu G. Tansey at Emory University, USA, and colleagues compared the levels and function of LRKK2 in immune cells from 40 late-onset PD patients and 32 age- and environment-matched healthy controls. The cells from PD patients had higher levels of LRKK2 protein and produced more pro-inflammatory molecules in response to stimulation than the control cells. As exacerbated inflammatory responses are known to aggravate neurodegeneration, monitoring LRKK2 levels may aid the assessment of disease progression in both inherited and sporadic cases of PD.

## Introduction

Parkinson’s disease (PD) is a progressive age-related movement disorder. The histopathological features of PD include degeneration of dopaminergic neurons in the substantia nigra pars compacta (SNpc) and the presence of Lewy bodies (neuronal inclusions of aggregated α-synuclein and other ubiquitinated proteins). Despite decades of extensive study, the etiology of the sporadic form of PD remains unclear. Thus, the development of new treatments and therapeutics has been slow-paced. In 2004, multiple labs identified mutations in the leucine-rich repeat kinase 2 (*LRRK2*) gene as causative for a dominantly inherited form of PD, leading to an exciting new pathway for researchers to pursue.^1, 2^ Mutations have also been found in sporadic cases at rates varying from 0.3–41% depending on the country of origin and ethnicity of the population studied.^3^ Due to similarities in the clinical presentation of LRRK2-associated PD and idiopathic PD,^3–5^ the study of LRRK2 function has the potential to offer new insight into the mechanisms underlying sporadic PD etiology.

The *LRRK2* gene is large, containing 51 exons that code for a 2527-amino acid protein of large molecular weight (~286 kDa) with several different functional and protein-interacting domains.^6, 7^ Enzymatic domains include a ROC (Ras of complex) GTPase domain^8^ and a serine/threonine kinase domain.^9^ There are also multiple protein-interacting domains, including a leucine-rich repeat domain, a C-terminal WD40 repeat domain, and armadillo and ankyrin repeat domains.^6, 7^ Given the multiple, highly diverse enzymatic and protein interacting domains, it is likely that LRRK2 may have different binding partners in different cell types. In support of this, LRRK2 has been shown in vitro to influence regulation of autophagy, macroautophagy,^10^ ceramide metabolism,^11^ neurite outgrowth, vesicular trafficking, cytoskeletal components, and cell signaling pathways involving nuclear factor of activated T cells (NFAT), Wnt, and nuclear factor-κB.^10, 12–17^

Multiple mutations and normal genetic variations in the *LRRK2* gene have been associated with disease.^18^ The six most common pathogenic mutations in LRRK2 associated with PD^19^ reside in the GTPase and kinase domains.^20, 21^ The most prevalent mutation is the G2019S mutation in the kinase domain.^3^ Although mutations in LRRK2 only account for 1–2% of all PD cases, they are particularly prevalent in individuals of Ashkenazi Jewish (29.7%) and North African Arab ancestry (41%) (ref. 3). The penetrance of the most common *LRRK2* mutation (G2019S) ranges from 28% at 54 years of age to 74% at 79 years of age, suggesting that genetic and environmental modifiers influence lifetime risk for PD in individuals with these mutations.

Importantly, LRRK2 is not only expressed in neurons but is also expressed in cells of both the innate and adaptive immune system.^22, 23^ Interestingly, LRRK2 polymorphisms have been associated with Crohn’s disease, an autoimmune inflammatory bowel disease, and leprosy, an infection caused by *Mycobacterium leprae*, supporting a link to immune function.^24, 25^ LRRK2 is also a member of the receptor interacting protein kinase family, which are proteins that detect and respond to cellular stress by regulating cell death and activation of the immune system.^26, 27^ Pro-inflammatory signals, such as interferon-γ (IFN-γ),^23, 28, 29^ lipopolysaccharide (LPS),^22, 30^ and interleukin (IL)-1β (ref. 17) have been shown to increase LRRK2 expression. Specifically, in CD14^+^ macrophages, CD3^+^ T cells, and CD19^+^ B cells, increases in LRRK2 following IFN-γ stimulation have been observed.^23, 28, 29^ Also, IFN-γ stimulation was shown to increase LRRK2 mRNA and protein expression in the non-classical CD14^+^CD16^+^ monocyte population.^28^ Pharmacological inhibition of LRRK2 with multiple inhibitors resulted in decreased CD14, CD16, and major histocompatibility complex (MHC-II) expression, suggesting that LRRK2 may play a significant role in the activation of monocytes via IFN-γ.^28^ In addition, use of LRRK2 inhibitors results in decreased LRRK2 protein expression after 24 h exposure in peripheral blood mononuclear cells (PBMCs).^31^ Recently, it was reported that increased expression of LRRK2 in monocytes following IFN-γ stimulation occurs via a mechanism involving extracellular signal-related kinase 5 (ERK5) signaling.^29^

Despite a growing wealth of evidence that LRRK2 is enriched in both innate and adaptive immune cells,^26, 27^ to date the majority of studies involving LRRK2 and its associated mutations have mainly been assessed for their effects on neuronal function. Given that increased inflammation in the periphery and the brain has been associated with the pathophysiology of PD,^32–36^ it is possible that LRRK2 may play a role in this process and serve as a regulator of inflammatory and immune responses that influence risk for age-related degeneration and risk for PD. Based on the current literature, we hypothesized that LRRK2 expression is increased in cells from PD patients, causing a dysregulation of function and activation in cells of both the innate and adaptive immune system. To test this, we investigated the extent to which various peripheral immune cell types express LRRK2 and inflammatory cytokines under resting conditions and during immune activation in healthy individuals and in patients with sporadic PD.

## Results

### Validation of Abcam c41-2 LRRK2 antibody for detection of human LRRK2 by flow cytometry

The c41-2 Abcam LRRK2 (MJFF2) antibody is a rabbit polyclonal antibody used in the studies herein to detect human LRRK2 protein in peripheral immune cell populations. Because the LRRK2 antibody was not conjugated to a fluorophore, a fluorescein isothiocyanate (FITC)-conjugated secondary anti-rabbit IgG antibody was used for the flow cytometry studies. Although the manufacturer notes that c41-2 is cross-reactive with mouse LRRK2 protein in western blotting and histological applications, c41-2 only detects mouse LRRK2 when it is highly overexpressed as in the mouse WT-LRRK2- or mouse G2019S-LRRK2-overexpressing BAC transgenic lines (Supplementary Fig. S1a). In our hands, c41-2 displays minimal cross-reactivity with the mouse LRRK2 protein relative to the human protein in peripheral blood immune cells under conditions for western blotting of sodium dodecyl sulfate polyacrylamide gel electrophoresis. Consistent with this, the flow signal from endogenous mouse LRRK2 protein in PBMCs from C57BL/6J mice is indistinguishable from that of PBMCs from LRRK2 KO mice (Supplementary Fig. S1b). In addition, there is a minimal shift in the histograms for the PBMCs from the mouse WT LRRK2- or mouse G2019S-LRRK2-overexpressing BAC transgenic mouse lines indicating lack of recognition of mouse LRRK2 via flow cytometry (Supplementary Fig. S1b).

**Fig. 1:**
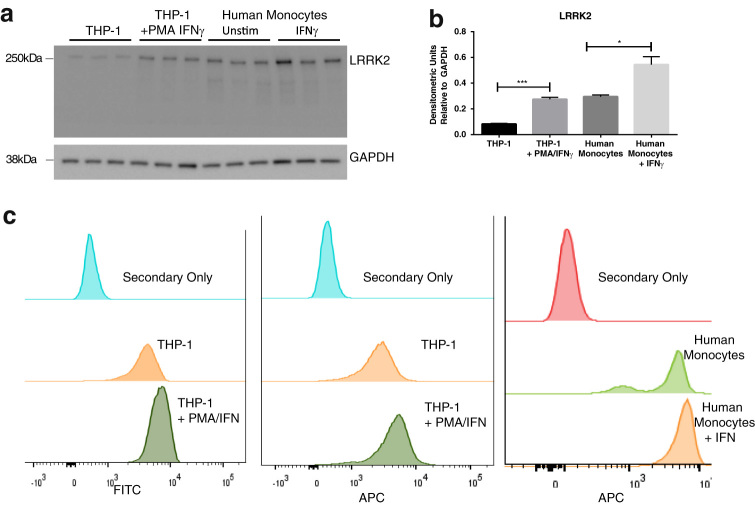
Antibody validation for detection of human LRRK2 protein by flow cytometry. **a**, **b** Increases in human LRRK2 protein in PMA differentiated/IFN-γ-stimulated THP-1 human monocytic cell line, IFN-γ-stimulated human monocytes from peripheral blood are detectable by western blot with the c41-2 LRRK2 antibody. Western blot analysis of LRRK2 levels in naive THP-1 (*n* = 3) and PMA-differentiated and IFN-γ-stimulated THP-1 cells (*n* = 3) compared to human primary monocytes from peripheral blood (*n* = 3). **c** Increases in human LRRK2 protein in permeabilized PMA differentiated/IFN-γ-stimulated THP-1 cells are detectable by flow cytometry with the c41-2 LRRK2 antibody and a FITC-conjugated secondary antibody (1:10,000) or an Alexa 647-conjugated secondary (1:10,000)

To validate the specificity of the LRRK2 antibody for human LRRK2 using flow cytometry applications, we performed experiments in which induction of the human LRRK2 protein was confirmed in both a human monocytic cell line (THP-1) and primary human monocytes using both western blot and flow cytometry under the same experimental conditions. A single immunoreactive band was detectable in immunoblots with the c41-2 LRRK2 antibody in T cells, B cells, and monocytes isolated from human peripheral blood (Supplementary Fig. S1a). Next, naive THP-1 cells were found to express low amounts of LRRK2 protein. Upon treatment with PMA, the cells differentiate into macrophages and following stimulation with IFN-γ, their expression of LRRK2 protein can be shown to increase by western blot (Fig. 1a, b). Importantly, this increase is accompanied by an increase in the LRRK2 signal by flow cytometry (Fig. 1c) using the c41-2 LRRK2 antibody and either of two different secondary antibodies (Alexa-Fluor647 or FITC). In addition, human primary monocytes isolated from peripheral blood were found to express detectable levels of human LRRK2 protein by western blotting with c41-2 (Fig. 1a, b) and by flow cytometry with the same LRRK2 antibody (Fig. 1c). As was seen in the cell lines, the levels of LRRK2 in the primary cells could be increased further by stimulation with IFN-γ as measured by western blot (Fig. 1a, b) and flow cytometry (Fig. 1c).

### PD is associated with increased LRRK2 expression in innate and adaptive immune cells

**Fig. 2:**
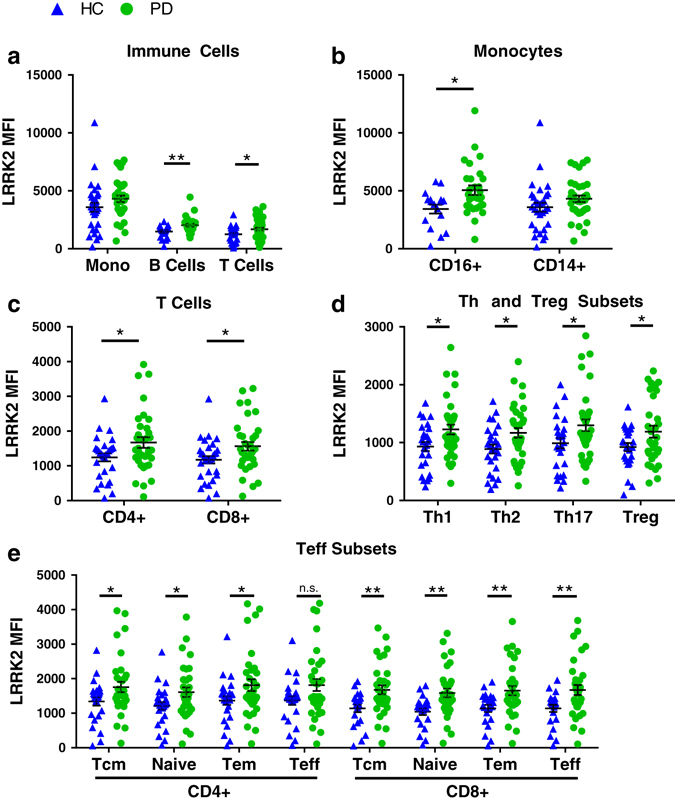
LRRK2 expression in T cells, B cells, and a subset of monocytes is increased in PD patients compared to matched HC subjects. **a** LRRK2 median fluorescence intensity (MFI) in monocytes (*t*(64) = 1.57, *p* = 0.122, HC *n* =  30, PD *n* = 36), B cells (*t*(47) = 3.02, *p* = 0.004, HC *n* = 21, PD *n* = 28), and T cells (*t*(63) = 2.35, *p* = 0.022, HC *n* = 29, PD *n* = 36), **b** a subset of monocytes (*t*(44) = 2.68, *p* = 0.01, HC *n* =  17, PD *n* = 29) **c**–**e** and T-cell subsets (CD4^+^, *t*(61) = 2.17, *p* = 0.034, HC *n* = 29, PD *n* = 34; CD8^+^, *t*(65) = 2.39, *p* = 0.02 HC *n* = 31, PD *n* = 36; T helper HC *n* = 27, PD *n* = 34: Th1, *t*(59) = 2.49, *p* = 0.016; Th2, *t*(59) = 2.41, *p* = 0.019; Th17, *t*(59) = 2.24, *p* = 0.029; Treg, *t*(55) = 2.06, *p* = 0.044 HC *n* = 26, PD *n* = 31; CD4^+^ effector subsets HC *n* = 26, PD *n* = 33: Tcm *t*(57) = 2.08, *p* = 0.043; Naive *t*(57) = 2.18, *p* = 0.033; Tem *t*(57) = 2.02, *p* = 0.049; Teff, *t*(57) = 1.99, *p* = 0.051; CD8^+^ effector subsets HC *n* = 22, PD *n* = 33: Tcm *t*(53) = 2.95, *p* = 0.005; Naive *t*(53) = 3.16, *p* = 0.003; Tem *t*(53) = 2.81, *p* = 0.007; Teff *t*(53) = 2.70, *p* = 0.009) was determined by flow cytometry staining of total peripheral blood mononuclear cells. Means were plotted with standard error of the mean. Two-tailed Student’s *t*-test between HC and PD was used to test for significance. *p < 0.05, **p < 0.01

To investigate if LRRK2 levels differ between specific immune cell populations and between healthy individuals and those with PD, immunophenotyping of peripheral blood was performed in cells from PD and age-matched healthy control (HC) subjects. LRRK2 expression has been reported in both monocytes and B cells,^22, 23^ however, there are conflicting reports as to whether LRRK2 is expressed in T cells.^22, 28^ To date, the most commonly used approach to ascertain LRRK2 expression levels has been western blotting and quantitative reverse transcriptase-polymerase chain reaction. Differences between antibodies and oligonucleotide primers could account for discrepancies between assays and labs. We found that LRRK2 protein levels, reported as median fluorescence intensity (MFI), were increased in CD16^+^ monocytes, T cells, and B cells from PD patients compared to HC (Fig. 2a, b). Importantly, the increase in LRRK2 is not a reflection of overall increases in protein content in immune cells in PD subjects vs. HCs as other cell-specific markers such as CD19, CD14, and 4-1BB were not significantly different between HC and PD subjects (Supplementary Fig. S2).

Given the diverse functions associated with T-cell subsets, we determined whether the increased LRRK2 levels in the PD group were altered in all subsets or only specific subsets. The data were stratified by CD8^+^ effector subsets and CD4^+^ effector, helper, and regulatory subsets (Supplementary Table 3). We found no significant difference in LRRK2 expression between different T-cell subsets, but its levels are globally increased in all subsets in PD patients compared to HC subjects (Fig. 2c–e). One small exception is the CD4^+^ Teff subset; when comparing the PD and HC subjects the *p*-value was 0.051. In our study cohort, patients with PD had increased LRRK2 expression in T cells, B cells and a pro-inflammatory subset of monocytes (CD16^+^).

**Fig. 3:**
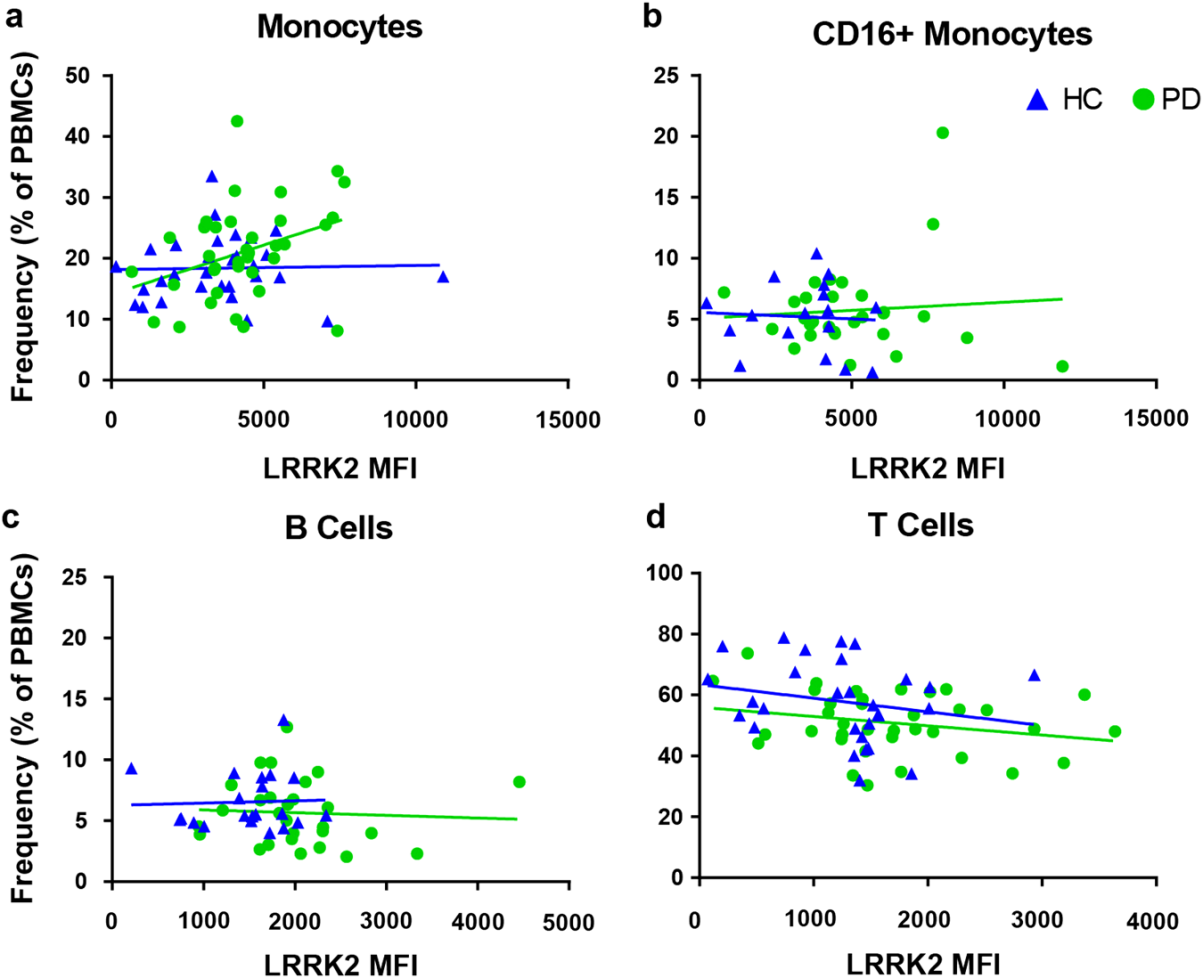
LRRK2 expression in monocytes positively correlates with monocyte frequency in PD patients but not in HC subjects. Frequency of **a** monocytes (HC *R* ^*2*^(30) = 0.001, *p* = 0.88; PD *R* ^*2*^(36) = 0.125, *p* = 0.034); *F*(1, 62) = 3.23, *p* = 0.077), **b** CD16^+^ monocytes (HC *R* ^*2*^(17) = 0.004, *p* = 0.822, PD *R* ^*2*^(29) = 0.006, *p* = 0.684; *F*(1,42) = 0.148, *p* = 0.703), **c** B cells (HC *R* ^2^(23) = 0.002, *p* = 0.860, PD *R* ^*2*^(28) = 0.003, *p* = 0.773; *F*(1, 45) = 0.092, *p* = *0.763*) and **d** T cells (HC *R* ^*2*^(29) = 0.045, *p* = 0.267, PD *R* ^*2*^(37) = 0.061, *p* = 0.146; *F*(1, 61) = 0.117, *p* = 0.734) as a percentage of total PBMCs determined by flow cytometry staining were plotted vs. LRRK2 median fluorescence intensity (MFI). Means were plotted with standard error of the mean. ANCOVA was performed to assess differences of slopes between HC and PD. Linear regression was used to assess individual correlations of slopes of HC and PD. Significance was set at *p* < 0.05

In addition to protein expression levels in immune cells, we assessed the frequencies of immune cell populations in our subjects. No significant differences between PD and HC in the frequencies of monocytes or B cells were observed (Supplementary Fig. S3a–b), but PD patients had a significantly decreased T-cell frequency compared to HC (Supplementary Fig. S3a). Consistent with what has been previously reported, it appears that this decrease is due strictly to the CD4^+^ subset and not to CD8^+^ T-cell frequencies^37^ (Supplementary Fig. S3c–e). In addition, there was a positive correlation between the expression level of LRRK2 and frequency of monocytes in PD patients (Fig. 3a). There was increased LRRK2 expression in subjects with higher frequencies of CD14^+^ monocytes. Although not reaching significance, there was a trend towards the correlations between LRRK2 expression and CD14^+^ frequencies between PD and HC being distinctly different (Fig. 3a). Other correlations between LRRK2 level and immune cell frequency were not statistically significant between PD and HC (Fig. 3b–d). In summary, LRRK2 expression is increased in lymphocytes and inflammatory monocytes from PD patients and the levels of LRRK2 positively correlate with the frequency of monocytes in PD patients.

### LRRK2 expression is induced by inflammatory stimuli in both PD and HC monocytes but shows opposite correlation with MHC-II induction in PD vs. HC subjects

**Fig. 4:**
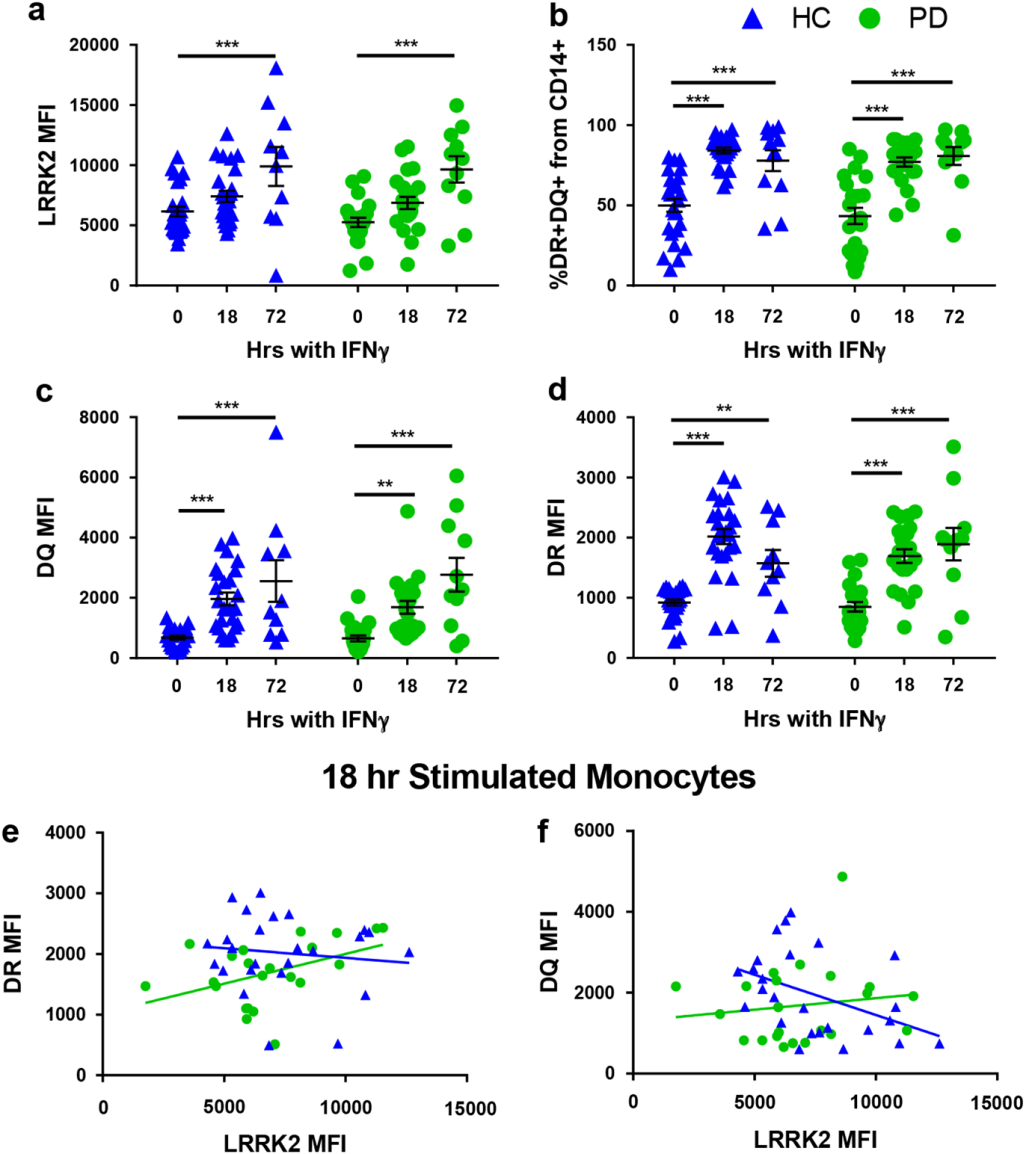
The IFNγ-stimulated increase in LRRK2 protein in PD and HC monocytes displays similar kinetics but the correlation between LRRK2 levels and MHC-II expression is different for PD vs. HC. **a** LRRK2 induction (HC *p* = 0.0006, PD *p* < 0.0001), **b** %DR^+^DQ^+^ monocytes (18 and 72 h HC *p* < 0.0001, PD *p* < 0.0001), **c** HLA-DQ induction (18 h HC *p* = 0.0001, PD *p* = 0.0042; 72 h HC *p* < 0.0001, PD *p* < 0.0001), and **d** HLA-DR induction (18 h HC *p* < 0.0001, PD *p* < 0.0001; 72 h HC *p* = 0.0049, PD *p* < 0.0001) with or without 100 U/mL interferon-γ stimulation for 18 (HC *n* = 25, PD *n* = 22) or 72 h (HC *n* = 10, PD *n* = 11) in paramagnetically, positively sorted monocytes was measured by flow cytometry. Means were plotted with standard error of the mean. Two-way ANOVA with Sidak’s multiple comparisons post-hoc test was used to test for significance. *p < 0.05, **p < 0.01, ***p < 0.001. **e** -DR median fluorescence intensity (MFI) (HC *R* ^*2*^(25) = 0.013, *p* = 0.580, PD *R* ^*2*^(22) = 0.195, *p* = 0.040; *F*(1,43) = 3.16, *p* = 0.083) and **f** -DQ MFI (HC *R* ^*2*^(25) = 0.193, *p* = 0.028, PD *R* ^*2*^(22) = 0.193, *p* = 0.540; *F*(1, 43) = 4.24, *p* = 0.046) were measured using flow cytometry and plotted against LRRK2 MFI. ANCOVA was performed to assess differences of slopes between HC and PD. Linear regression was used to assess individual correlations of slopes of HC and PD. Significance was set at *p* < 0.05

There are several reports indicating that LRRK2 expression in immune cells increases following inflammatory stimuli such as IFN-γ or microbial components like LPS.^22, 23, 30, 38^ We sought to replicate these data in monocytes and explore whether this paradigm held true in T cells. Cells were plated immediately following isolation from peripheral blood. Monocytes were stimulated with IFN-γ for 18 or 72 h. After18 h of stimulation, LRRK2 expression in monocytes was not significantly increased (Fig. 4a). However, by 72 h after stimulation, LRRK2 levels in monocytes were significantly increased relative to baseline in both PD and HC populations (Fig. 4a). MHC-II, also known as human leukocyte antigen (HLA), is the antigen-presenting-molecule expressed on cells such as monocytes that activates CD4^+^ T cells and is upregulated following an inflammatory stimulus. In humans, there are three different isotypes, HLA-DR, -DQ, and -DP, encoded by the MHC-II locus.^39^ To determine if there were any alterations in antigen presentation in patients with PD, we assessed expression levels of HLA-DR and -DQ. Both PD and HC groups displayed induction of HLA-DR and -DQ proteins after IFN-γ stimulation with 80–90% of cells being HLA-DR/-DQ double positive (Fig. 4b). Monocytes from both HC subjects and PD patients continued to upregulate HLA-DQ over time (Fig. 4c). Both groups also upregulated HLA-DR over time; however, HC subjects downregulated HLA-DR after 72 h while monocytes from PD patients retained expression (Fig. 4d). Furthermore, after 18 h of stimulation, LRRK2 levels were positively correlated with HLA-DR MFI (Fig. 4e) in monocytes of PD subjects and negatively correlated with HLA-DQ MFI (Fig. 4f) in monocytes from HC patients. In summary, LRRK2 protein expression was induced in monocytes from both groups after stimulation with IFN-γ at both 18 and 72 h post-stimulation. However, LRRK2 expression was positively correlated with MHC-II induction in PD patients and negatively correlated in HC subjects.

### LRRK2 induction is slower in T cells compared to monocytes in both PD and HC subjects

**Fig. 5:**
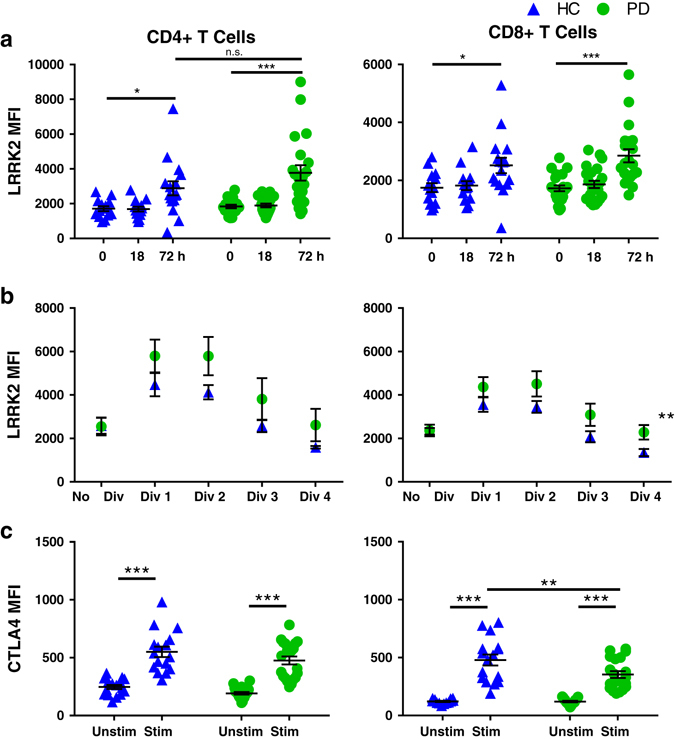
The anti-CD3/CD28-stimulated increase in LRRK2 protein in T cells is slower than in monocytes and similar in PD and HC subjects but T-cell proliferation is associated with greater increases in LRRK2 protein and dampened expression of the negative regulator of T-cell activation CTLA4 in PD subjects. LRRK2 median fluorescence intensity (MFI) of **a** stimulated cells (HC 18 h *n* = 14, 72 h *n* = 16, *p* = 0.019; PD 18 h *n* = 22, 72 h *n* = 20, *p* < 0.0001; HC to PD at 72 h, *p* = 0.0809), **b** proliferating cells (CellTrace Violet) (*p* = 0.0045, HC *n* = 12, PD *n* = 11) and **c** CTLA-4 MFI (HC *n* = 16, PD *n* = 20, CD8^+^ *p* = 0.0034) were measured using flow cytometry following stimulation of paramagnetically, positively selected T cells with 30 U/mL IL-2 and anti-CD3/CD28 stimulation beads for 72 h. Means were plotted with standard error of the mean. Two-way ANOVA with Sidak’s multiple comparisons post-hoc test was used to test for significance. *p < 0.05, **p < 0.01, ***p < 0.001

To determine the timing of LRRK2 induction following T-cell activation, T cells were stimulated with anti-CD3/CD28-coated beads and IL-2 (a cytokine necessary for T-cell activation and proliferation) for 18 or 72 h. Stimulation for 18 h was not sufficient to induce increases in LRRK2; however, LRRK2 levels after 72 h of stimulation were significantly increased in both CD4^+^ and CD8^+^ T cells in PD and HC groups. There were no significant differences between the means of LRRK2 expression in CD4^+^ T cells in PD and HC after 72 h stimulation (Fig. 5a).

### PD is associated with higher LRRK2 induction in proliferating T cells compared to HCs

To determine if LRRK2 may be involved in regulation of T-cell proliferation, cells were stained with CellTrace Violet, a cell proliferation dye, and analyzed via flow cytometry. Typically, after 72 h of stimulation T cells will have divided between three and four times. To simplify this analysis, each cell division was analyzed individually for LRRK2 expression. We found that LRRK2 was upregulated only in the early dividing cells in both PD and HC groups (Fig. 5b). In CD8^+^ T cells, dividing cells from PD patients had significantly higher levels of LRRK2 compared to dividing cells from HC subjects (Fig. 5b). Importantly, no differences were observed in the percent of CD4^+^ or CD8^+^ T-cell proliferation or percent of T cells that divided three times between PD and HC subjects (Supplementary Fig. S4a). Although the relationship between LRRK2 MFI and percentage of proliferated CD4^+^ T cells was not significantly different between the two groups, the LRRK2 MFI in CD8^+^ T cells was higher in PD patients than in HC subjects at every percent of proliferated cells (Supplementary Fig. S4b).

### The T-cell activation marker CTLA4 is significantly decreased in T cells of PD patients following stimulation

In addition to proliferation, we also assessed potential differences in activation markers normally upregulated following stimulation in T cells. If LRRK2 has a role in regulating processes involved in immune cell activation, increased expression levels of LRRK2 should correlate with differential activation of T cells. We looked at two common activation markers:CTLA-4, a negative regulator of activation, and 4-1BB, a receptor that amplifies T-cell activation by inducing secretion of IL-2. CTLA-4 competes with CD28 to bind CD80/86 on antigen-presenting cells; but while CD28 sends an activating signal when bound, CTLA-4 sends an inhibitory signal when bound. After the 72 h stimulation, both PD and HC T cells upregulated 4-1BB (Supplementary Fig. S2) and CTLA-4, however, CTLA-4 levels in CD8^+^ T cells of PD patients were significantly lower than those in HCs (Fig. 5c).

### Immune cells from PD patients display similar cellular cytokine expression but increased pro-inflammatory cytokine secretion

**Fig. 6:**
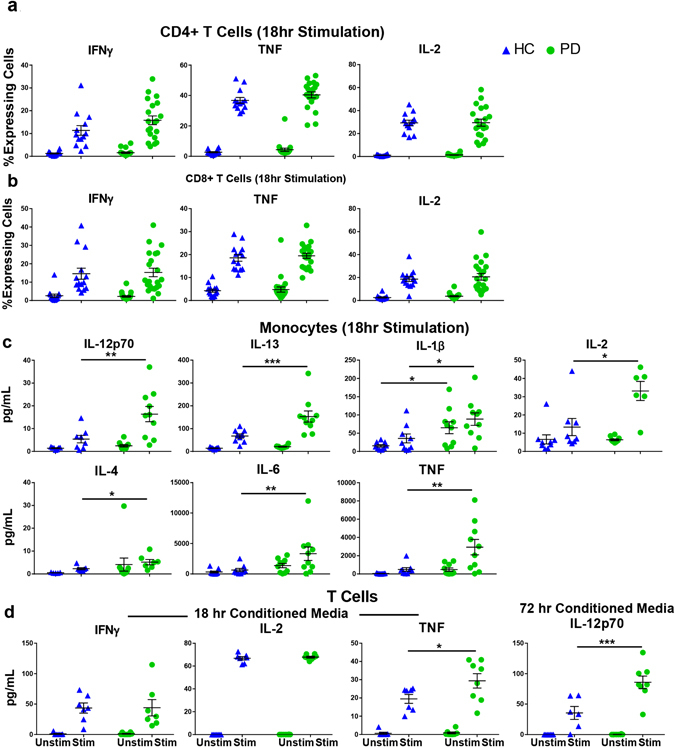
Monocytes and T cells from PD patients display increased cellular expression and secretion of pro-inflammatory cytokines in association with higher LRRK2 protein expression. **a**, **b** Cytokine expression measured through intracellular cytokine staining measured with flow cytometry (HC *n* = 13, PD *n* = 22), **c** multiplexed immunoassay on conditioned media from 18 h stimulated monocytes (HC *n* = 9, PD *n* = 10) and **d** 18 h (HC *n* = 7, PD *n* = 8, *p* = 0.0154) and 72 h (HC *n* = 7, PD *n* = 8, *p* = 0.0001) stimulated T cells. Means were plotted with standard error of the mean. Two-way ANOVA with Sidak’s multiple comparisons post-hoc test was used to test for significance. *p < 0.05, **p < 0.01, ***p < 0.001

We wanted to further explore a potential role for LRRK2 in immune cell activation. Following stimulation, T cells secrete cytokines to further activate the immune cell response.^40^ To investigate the hypothesis that LRRK2 expression affects cytokine production, we used intracellular cytokine staining (ICS) to measure cytokine expression in a cell type-specific manner as well as multiplexed immunoassays to measure cytokine secretion into the culture media. Cytokine measurements were performed after 18 h of stimulation. Prior to harvest, cells were treated with Brefeldin A, a compound that inhibits protein transport causing vesicle accumulation at the golgi complex/endoplasmic reticulum.^41^ Cells were stained to measure total intracellular protein expression of IFN-γ, tumor necrosis factor (TNF), and IL-2. Protein levels of all cytokines were increased with stimulation, but no significant differences were detected between PD and HC groups (Fig. 6a, b).

Multiplexed immunoassays were performed on conditioned media from the 18 h-stimulated monocyte and 18- and 72 h-stimulated T-cell samples to measure the levels of cytokines secreted into the conditioned media. For 18 h-stimulated monocytes, cytokine secretion was significantly increased in the PD group for IL-12p70, IL-13, IL-1β, IL-2, IL-4, IL-6, and TNF (Fig. 6c). In 18 h-stimulated T cells, no significant differences were detected in levels of secreted IFN-γ and IL-2, but T cells from PD patients secreted significantly more TNF compared to cells from HC subjects (Fig. 6d). Interestingly, at 72 h, IL-12p70 was the only cytokine secreted at higher levels in PD T cells compared to HC T cells (Fig. 6d). In summary, several immune cell subsets from PD patients secreted higher cytokines upon stimulation compared to immune cells from HC subjects.

### LRRK2 expression is positively correlated with cytokine expression in PD patients

**Fig. 7:**
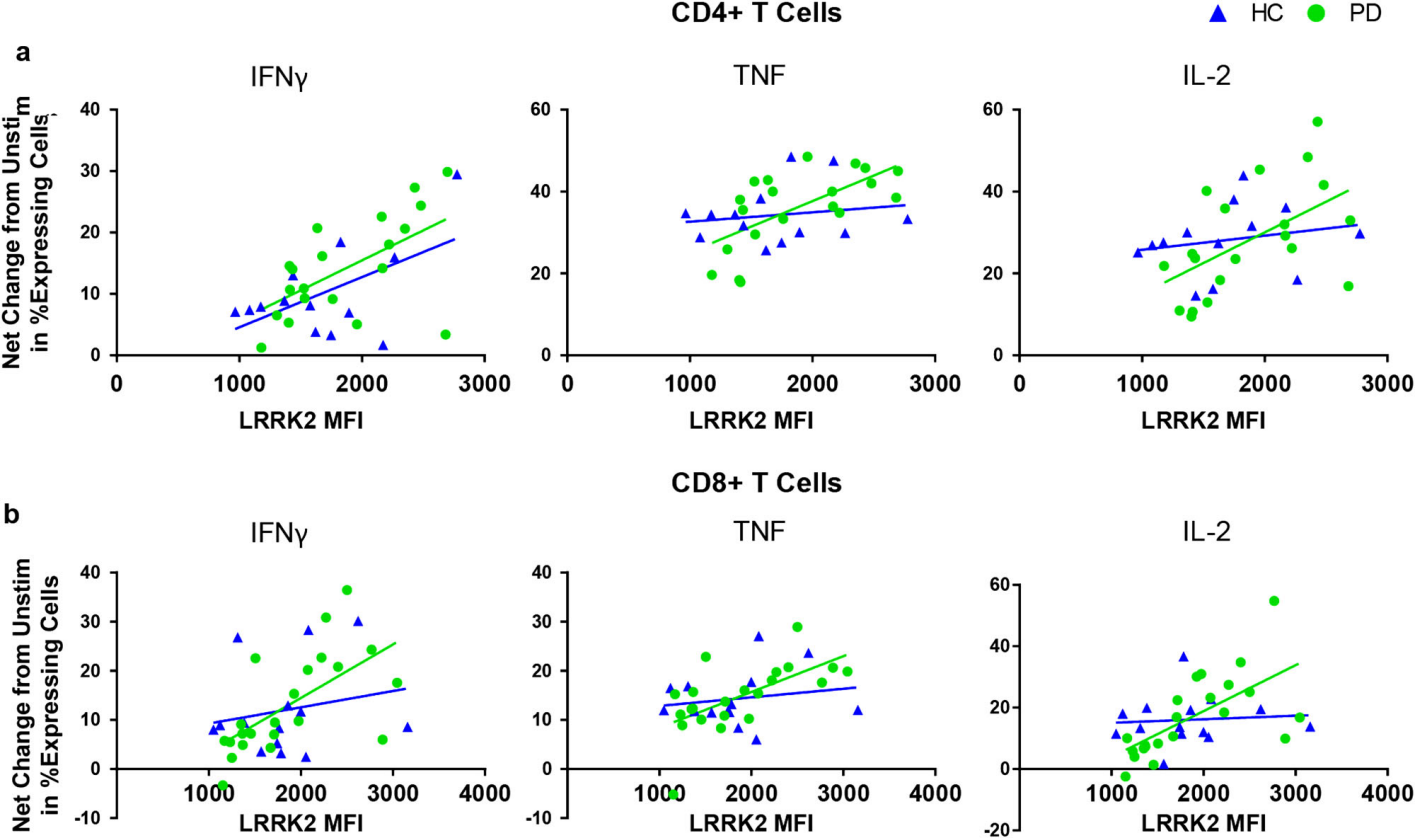
LRRK2 protein in stimulated CD4^+^ and CD8^+^ T cells from PD patients displays significant positive correlations with cytokine-expression. Fold change in percent of cytokine secretion (IFN-γ, TNF, and IL-2) by **a** CD4^+^ T cells (IFN-γ: PD *R* ^*2*^(20) = 0.339, *p* = 0.002, HC *R* ^*2*^(13) = 0.307, *p* = 0.049; *F*(1, 29) = 0.106, *p* = 0.747;TNF: PD *R* ^*2*^(20) = 0.421, *p* = 0.002, HC *R* ^*2*^(13) = 0.028, *p* = 0.585; *F*(1, 29) = 3.63, *p* = 0.067; IL-2: PD *R* ^*2*^(20) = 0.293, *p* = 0.0014, HC *R* ^*2*^(13) = 0.044, *p* = 0.494; *F*(1, 29) = 2.19, *p* = 0.150) and **b** CD8^+^ T cells (IFN-γ: PD *R* ^*2*^(22) = 0.400, *p* = 0.002, HC *R* ^*2*^(14) = 0.040, *p* = 0.494; *F*(1, 32) = 2.04, *p* = 0.163; TNF: PD *R* ^*2*^(22) = 0.398, *p* = 0.002, HC *R* ^*2*^(14) = 0.031, *p* = 0.549; *F*(1, 32) = 2.65, *p* = 0.113; IL-2: PD *R* ^*2*^(22) = 0.430, *p* = 0.001, HC *R* ^*2*^(14) = 0.007, *p* = 0.780; *F*(1, 32) = 5.46, *p* = 0.026) relative to unstimulated cells plotted against LRRK2 median fluorescence intensity (MFI) of stimulated cells. ANCOVA was performed to assess differences of slopes between HC and PD. Linear regression was used to assess individual correlations of slopes of HC and PD. Significance was set at *p* < 0.05

PD patients had higher LRRK2 expression in cells with a higher amount of intracellular cytokine levels (Fig. 7a, b). T cells from PD patients displayed an association between LRRK2 protein levels and cytokine expression (Fig. 7a, b). LRRK2 protein levels positively correlated with IFN-γ, TNF, and IL-2 expression in T cells from PD patients; however, only IFN-γ expression in CD4^+^ T cells correlated with LRRK2 protein levels in HC subjects (Fig. 7a). In addition, when comparing the two subject groups for interaction, only the correlation between IL-2 expression in CD8^+^ T cells and LRRK2 MFI displayed significantly different slopes (Fig. 7b). In summary, LRRK2 levels positively correlated with cytokine expression and secretion in PD but not HC subjects.

## Discussion

Recent new discoveries in immune cells have suggested a potential link for LRRK2 to the regulation of the immune system and modulation of inflammatory responses. Herein, we demonstrate that LRRK2 is expressed in both innate and adaptive human immune cells and is expressed at higher levels in the immune cells of patients with late-onset PD compared to age-matched HC individuals. In addition, LRRK2 is induced in both human monocytes and T cells following an inflammatory stimulus; and in PD patients there is a positive correlation between LRRK2 levels, MHC-II induction, cytokine expression and secretion levels, and dampened expression of the T-cell inhibitory factor CTLA-4. Given the established role of LRRK2 as a potential negative regulator of NFAT,^16^ the differences we observed between PD patients and HC subjects suggest that LRRK2 may be important in the regulation of both innate and adaptive immune cells within the context of PD. Of note, within our study population and similar to other study populations, the PD group was predominantly male while the HC group was predominantly female as they were generally the spouses and caregivers of the PD patients. To ensure that the differences we observed were not due to this unequal sex distribution, results were stratified by sex but did not affect the differences observed between PD and HC groups.

The increased levels of LRRK2 associated with PD suggest that the protein is contributing to disease pathogenesis. CD14^+^CD16^+^ monocytes are considered to represent a non-classical monocyte population that is characteristically more pro-inflammatory and typically display increased cytokine secretion and greater antigen presentation.^42^ Therefore, the finding that LRRK2 levels are notably increased in CD16^+^ monocytes of PD patients may have functional relevance to disease risk or progression given that it was recently reported that IFN-γ induces more LRRK2 in this subset of monocytes compared to classical CD16^—^ monocytes.^28^ In addition, it has been shown that circulating monocytes are dysregulated in PD, displaying a hyperactive inflammatory phenotype.^43^ The monocyte data taken together with the increased LRRK2 expression in T cells and B cells suggest that following an inflammatory challenge, individuals with PD will mount an exacerbated inflammatory response compared to healthy individuals that could lead to sustained immune activation and acceleration of disease progression.

In support for the role of LRRK2 in immune cell regulation, we found that LRRK2 protein expression increases in human immune cells following inflammatory challenges. Thereafter, LRRK2 levels continue to increase over time in monocytes. Monocytes are antigen-presenting cells that are critical to the activation of the adaptive immune response by upregulating MHC-II (HLA-DR/-DQ) proteins loaded with antigens that activate CD4^+^ T cells. Although we observed no differences in the overall extent of MHC-II induction between the PD and HC groups, in HC subjects, LRRK2 expression is negatively correlated with HLA-DQ expression whereas in PD patients, LRRK2 expression is positively correlated with HLA-DR expression. These data suggest that LRRK2 could be regulating the antigen presentation function of human monocytes, and that such regulation is altered in PD patients. Studies have shown that inhibition of LRRK2 kinase activity with the LRRK2-IN1 inhibitor decreased expression of CD14, CD16, and MHC-II in monocytes.^28^ However, due to the reported off-target effects of LRRK2-IN1, specifically ERK5 inhibition,^44^ it is possible that this decrease cannot be attributed wholly to LRRK2 kinase activity. Alternatively, it is possible that LRRK2 localizes and associates with binding partners involved in regulation of antigen presentation and that LRRK2 kinase activity is not required for such interactions. In addition, recent studies suggest that antigen presentation is altered in a subset of individuals with PD in association with a non-coding SNP (*rs 3129882*) in the MHC-II locus that synergizes with environmental exposures to increase risk for PD.^45^ Although the LRRK2 and MHC-II loci are encoded on different chromosomes, perhaps the mechanism of altered antigen presentation increases susceptibility for sporadic PD.

Previous studies have declared LRRK2 absent or undetectable in T-cell populations,^28^ however, the data presented here definitively demonstrate inducible expression of LRRK2 not just in CD3^+^ T cells, but also in all functional T-cell subsets. There were no significant differences between baseline levels of LRRK2 in subsets of T cells; however, LRRK2 levels were increased in all of the T cells of PD patients relative to those of HC subjects. Given that LRRK2 has been shown to be a negative regulator of NFAT in HEK293 T cells,^16^ and NFAT is a necessary transcription factor for T-cell activation, we hypothesize that LRRK2 could also serve as a negative regulator of T-cell activation via interaction with NFAT. Induction of LRRK2 was also seen in T cells, but was delayed until 72 h and only seen in dividing T cells. PD patients also had greater upregulation of LRRK2 in these dividing T cells, raising the interesting possibility that LRRK2 is essential for T-cell division or acting in some sort of regulatory capacity during T-cell division.

Markers of T-cell activation such as CTLA-4 and 4-1BB are good indicators of proper immune response and regulation. Immune cells upregulate these markers following stimulation to keep the inflammatory response in check. Interestingly, T cells from PD patients have an impaired capacity to upregulate CTLA-4 compared to those from HCs. CTLA-4 transcription is controlled by NFAT1 binding to the proximal promoter and is decreased when NFAT is inhibited.^46^ Therefore, our findings suggest that in healthy individuals LRRK2 is a negative regulator of T-cell activation. Specifically, the increase in LRRK2 induced by stimulation acts to limit NFAT-dependent transcription, as evidenced by reductions in expression of CTLA-4. In PD patients, alterations in LRRK2 function may translate into poor regulation of T-cell responses and result in a pro-inflammatory environment characteristic of PD.

Cytokine secretion by T cells is a necessary step for promoting the immune response, but prolonged secretion and/or increased cytokine levels are hallmarks of inflammatory disease. In the CSF and nigrostriatal regions of PD brains examined at autopsy, the levels of pro-inflammatory cytokines such as IL-1β, TNF, IFN-γ, and IL-6 were increased compared to those of age-matched HC subjects.^47–49^ In the present studies, monocytes and T cells from PD patients were shown to secrete more pro-inflammatory cytokines than T cells from healthy individuals. In addition, both CD4^+^ and CD8^+^ T cells of PD patients expressing higher levels of inflammatory cytokines also expressed higher levels of LRRK2 suggesting that LRRK2 protein expression is associated with the inflammatory response characteristically seen in this neurodegenerative disease. Taken together, our findings suggest that LRRK2 levels in peripheral T cells may serve as a biomarker for diagnosis and monitoring disease progression in PD patients.

The significance of ourfindings are threefold. First, the selective induction of LRRK2 in pro-inflammatory monocytes and its potential regulatory role in antigen presentation is a crucial step in activation of the immune response and merits further exploration. Second, defining the kinetics of LRRK2 expression and its regulation in activated T cells is a critical first step toward understanding the role that LRRK2 plays in the adaptive immune system and its potential link to PD pathogenesis. Third, the positive correlations between LRRK2 expression levels in T-cell subsets, cytokine expression and secretion, and T-cell activation states suggest that targeting LRRK2 with therapeutic interventions is likely to have direct effects on immune cell function—whether this affords benefit or untoward bystander effects remains to be determined and is prerequisite to advancing LRRK2 kinase inhibitors to clinical trials.

## Materials and methods

### Human subjects

PD patients (40) and age-matched HCs (HC) (32) subjects were recruited through the Immune System and Neurological Disease (ISND) Institutional Review Board (IRB)-approved research protocol at the Emory Movement Disorders Clinic and community outreach events. Subjects were excluded based on age (younger than 50 and over 85 years of age), known familial PD mutations and/or other known neurological, chronic or recent infections, or autoimmune comorbidities. Subjects were genotyped for the G2019S LRRK2 mutation (LifeTechnologies #4351378, Grand Island, NY).

During recruitment, a confidential family history and environmental questionnaire was used to assess history of disease and inflammation/immune-relevant environmental exposures and comorbidities. Caffeine, nonsteroidal anti-inflammatory drug, and nicotine exposure was calculated as milligram-years, milligram-years, and pack-years, respectively. The study populations were balanced with respect to risk factors for PD,^50–53^ including age, smoking, nonsteroidal anti-inflammatory drug use, caffeine intake, and rs3129882 (HLA-DRA SNP) genotype (Supplementary Table 1). Study population was not sex-matched: the PD group was predominantly male while HC subjects tended to be more female as they were generally the caregivers; however, when the results are stratified by sex, sex does not account for the differences observed. Clinical severity of PD symptoms was also assessed using Hoehn and Yahr ratings and part II and III of the Unified Parkinson’s Disease Rating Scale.

### Human PBMC isolation, stimulation, and purification

Isolation, stimulation, and purification of peripheral immune cells was performed as previously published in Kannarkat et al.^45^. Briefly, PBMCs were isolated from whole blood using density centrifugation with Ficoll-Paque (GE Healthcare, Uppsala, Sweden). Monocytes and T cells were isolated from PBMCs using positive selection columns with anti-CD14 and anti-CD3 paramagnetic beads, respectively (Miltenyi Biotec, Bergisch Gladbach, Germany). The isolated cell fraction was processed for flow cytometry as follows. Monocytes and T cells were plated for stimulation in a 12-well plate at a density of 1x10^6^ cells per well. Monocytes were plated for 18 or 72 h with or without 5 ng/mL IFN-γ (PeproTech, Rocky Hill, NJ, USA). T cells were stimulated for 18 or 72 h with or without anti-CD3/CD28 beads (1:1 cells:beads) (Dynabeads® Life Technologies, Grand Island, NY, USA) and 30 U/mL recombinant human IL-2 (Biolegend, San Diego, CA, USA).

### Mouse PBMC isolation

The following mouse strains were obtained from Jackson labs:B6.Cg-Tg(Lrrk2*G2019S)2Yue/J (#012467), B6.Cg-Tg(Lrrk2)6Yue/J (#012466), C57BL/6J (#000664), and C57BL/6-Lrrk2^tm1Mjfa^/J (#012444). Blood from each mouse (200 µL) was collected in an EDTA vacutainer tube (BD Biosciences) via cheek bleed. Blood was incubated in the dark for 10 min at room temperature with 1x RBC lysis buffer (Biolegend) to lyse red blood cells. Cells were pelleted and resuspended in phosphate buffered saline (PBS) for flow cytometry processing or lysed in sample buffer for western blot as detailed below.

### Human THP-1 monocytic cell culture and differentiation

THP-1 cells (ATCC #TIB-202) were maintained in RPMI1640 medium with 2 mM L-glutamine adjusted to contain 1.5 g/L sodium bicarbonate, 4.5 g/L glucose, 10 mM HEPES, and 1 mM sodium pyruvate and supplemented with 10% fetal bovine serum. THP-1 cells were terminally differentiated into macrophages by exposure to 100 nM of phorbol 12-myristate 12- acetate (PMA) (Sigma P-8139) for 72 h. Terminal differentiation was confirmed as cells become adherent. Stimulation with 200 U/ml IFN-γ for 18 h was performed to further increase LRRK2 protein levels. Trypsin was used to lift the differentiated cells prior to use in western blotting or flow cytometry. For flow cytometry, cells were permeabilized and stained intracellularly with Abcam c41-2 Rb anti-LRRK2 antibody (1:50) and either a FITC-conjugated secondary (1:10,000) or an AlexaFluor-647-conjugated secondary (1:10,000).

### Western blotting

THP-1 cells and human monocytes were lysed in 4x sample buffer (Bio-Rad, Hercules, CA, USA) and heated at 80 °C for 5 min proteins were then separated in a 12% polyacrylamide gel (Bio-Rad) by gel electrophoresis and transferred to a 45 µm polyvinylidene difluoride (PVDF) membrane (Sigma Aldrich, St Louis, MO, USA). Following transfer, the membrane was cut along the pre-stained standard band indicating 100 kDa molecular weight and incubated in a 5% skim milk blocking solution in 1x tris-buffered saline containing 0.1% Tween-20 (TBST) (Sigma Aldrich). The corresponding membrane sections were incubated overnight in blocking solution containing a primary antibody targeting either GAPDH (SC-31915 1:1000; Santa Cruz Biotechnology, Dallas, TX, USA) or LRRK2 (MJFF c41-2, 1:5000; Abcam, Cambridge, UK) at 4 °C. Following three 5-min washes in 0.1% TBST with agitation, membrane sections were incubated for 1 h at room temperature in either goat anti-rabbit (LRRK2) or rabbit anti-goat (GAPDH) horseradish peroxidase conjugated secondary antibodies (1:1000; Jackson ImmunoResearch, West Grove, PA, USA) in blocking solution. Membranes were again washed in 0.1% TBST, and imaged using 1:1 dilute SuperSignal West FemtoChemiluminescent Substrate (Thermo Scientific, Waltham, MA, USA). Band intensity was determined using ImageStudio Software (Li-Cor Biosciences, Lincoln, NE, USA). LRRK2 expression (~286 kDa) has been normalized to corresponding GAPDH (Fig. 1b; ~37 kDa).

Mouse PBMCs and T cells, B cells, and monocytes isolated directly from human blood were lysed in 1x Laemli buffer (40 mM NaF, 5% dithiothreitol, 1x phosphatase inhibitors, 1x protease inhibitors). Proteins were separated in a 7.5% polyacrylamide gel (Bio-Rad, Hercules, CA, USA) by gel electrophoresis and transferred to 45 µM PVDF membrane (Sigma Aldrich, St Louis, MO, USA). Following transfer, the membrane was cut below the pre-stained 75 kDa molecular weight and incubated in 5% milk blocking solution in 1x Tris-buffered saline containing 0.1% Tween-20 (Sigma Aldrich, St Louis, MO, USA) for 1 h. The corresponding membrane sections were incubated overnight in blocking solution containing primary antibody targeting either LRRK2 (MJFF c41-2, 1:5000; Abcam, Cambridge, UK) or β-actin (sc-47778 β-Actin (C4) HRP 1:10,000, Santa Cruz Biotechnology) at 4 °C. Following three 5-min washes in 1x TBS with 0.1% Tween-20 with agitation, the LRRK2 probed membrane section was incubated overnight in donkey anti-rabbit horseradish peroxidase conjugated secondary antibody (1:5000; Jackson ImmunoResearch, West Grove, PA, USA) in blocking solution at 4 °C. Membranes were washed 3 × 5 min in 1x TBS with 0.1% Tween-20, and imaged using Luminata Crescendo Chemiluminescent Substrate (Thermo Scientific).

### Flow cytometry analysis

To stain for flow cytometry, 5 × 10^5^ cells per well were washed once with PBS and incubated for 30 min at 4 °C with LIVE/DEAD Fixable Red (Life Technologies). Cells were incubated in 1x FACS buffer (1% bovine serum albumin, 0.1% sodium azide, and 1 mM EDTA) for 15 min at 37 °C with anti-human CCR7:phycoerythrin. Cells were washed and incubated for 20 min at 4 °C with surface antibodies detailed in Supplementary Table 2. Cells were intracellularly stained using the Fixation/Permeabilization Stain Kit per manufacturer’s protocol (eBiosciences, San Diego, CA, USA). ICS was performed on T cells stimulated for 18 h. Cells were incubated with 5 µg/mL Brefeldin A (Biolegend) for 7 h prior to harvest. After harvest, cells were processed according to protocols detailed above. Cells were run immediately on an LSRII instrument (BD Biosciences, Franklin Lakes, NJ, USA). Supra Rainbow Spherobeads (SpheroTech, Lake Forest, IL, USA) and OneComp Beads (eBiosciences) were used to set voltages and compensation settings between cytometry runs. Analysis of flow cytometry data was performed using FlowJo Software v10.X (Ashland, OR, USA). Gates were set according to Supplementary Fig. S5. Because the LRRK2 antibody is unconjugated, a secondary antibody only control condition was used to determine nonspecific binding and select for positively stained populations. Conjugated isotype control antibodies were used for the rest of the cellular protein markers stained. Fluorescence minus one controls were used to account for fluorescence spillover between channels. Protein expression levels are reported as MFI.

### Cell proliferation assays

To assess T-cell proliferation, cells were stained with CellTrace Violet (Life Technologies) according to the manufacturer’s protocol and plated for 72 h with anti-CD3/CD28 Dynabeads® and 30 U/mL recombinant human IL-2. Unstimulated cells were harvested at 18 h post-plating as cells do not remain viable for 72 h without stimulation. At harvest, Dynabeads® were removed using magnetic separation, washed, and stained according to the above flow analysis protocol.

### Meso scale discovery multiplexed immunoassays

Conditioned media from plated cells was collected during cell harvest and stored at -80 °C until sample analysis. Media analyte levels were measured in duplicate using 3-plex (IL-2, TNFα, and IFN-γ) and 10-plex (IFN-γ, IL-10, IL-12p70, IL-13, IL-1β, IL-2, IL-4, IL-6, IL-8, and TNFα) plates on a Sector 2400 instrument (Meso Scale Discovery, Rockville, MD, USA).

### Statistical analyses

A two-tailed Student’s *t*-test was used to make comparisons between HC subjects and PD patients in the immunophenotyping studies. A two-way analysis of variance (ANOVA) followed by Sidak’s multiple comparisons post-hoc test was used to compare baseline characteristics of the study population and inducibility of immune response following IFN-γ stimulation in monocytes or anti-CD3/CD28 stimulation in T cells. Data was plotted with means and standard error of the mean. Analysis of covariance (ANCOVA) was used to assess differences in slopes of correlations between HC and PD. Linear regressions were performed to assess correlations of individual slopes of HC and PD. All statistical tests used are indicated in the figure legends. Graphpad Prism Version 6.05 (Prism, La Jolla, CA, USA) software was used to perform all statistical analyses.

### Human subjects research approval

All procedures involving human subjects were approved by the IRB of Emory University in Atlanta, Georgia before study commenced. All participants provided written informed consent and the terms and risks of the study were thoroughly explained before inclusion in the study.

## Electronic supplementary material

Supplementary Tables

Supplementary Figure Legends

Supplementary Figure S1

Supplementary Figure S2

Supplementary Figure S3

Supplementary Figure S4

Supplementary Figure S5
