# Supplementary material for: LRRK2 levels in immune cells are increased in Parkinson’s disease
Source: NPJ Parkinsons Dis. 2017 Mar 28;3:11. doi: 10.1038/s41531-017-0010-8 (PMC5459798; doi:10.1038/s41531-017-0010-8)
Supplement: Supplementary file 1 — Supplementary Tables [file 41531_2017_10_MOESM1_ESM.docx]

**Supplementary Tables**

**Supplementary Table 1. Demographics of the Study Population.** Healthy Controls

(HC) and Parkinson’s disease (PD) subjects are matched for age, *rs3129882* HLA-DRA genotype, smoking (pack-yrs), caffeine (mg-yrs), NSAID use (mg-yrs), and head injuries.

Although the groups are not balanced for sex, these differences do not account for the differences seen in the data.

|  | **HC** | **PD** | **p-value** |
| --- | --- | --- | --- |
| **N** | 32 | 40 |  |
| **Age** | 65.7±1.86 | 67.4±1.32 | 0.46 |
| **Sex** | 24F, 8M | 11F, 29M | 0.0004 |
| **rs3129882 genotype** | 14AA, 9GG, 9AG | 15AA, 13GG, 12AG | 0.86 |
| **Smoking (pack-yrs)** | 2.39±1.1 | 5.88±13.4 | 0.17 |
| **Caffeine (mg-yrs)** | 10935±1380 | 12026±1606 | 0.62 |
| **NSAID Use (mg-yrs)** | 1018±180 | 981.5±284 | 0.92 |
| **Head Injuries** | 0.125±0.098 | 0.45±0.143 | 0.08 |

**Supplementary Table 2. Fluorophore-conjugated antibodies used for flow staining and their dilutions.**

| **Marker** | **Fluorophore** | **Dilution** | **Company** | **Catalog Number** |
| --- | --- | --- | --- | --- |
| LRRK2 | Unconjugated | 1:50 | abcam | ab133474 |
| F(ab')2 anti-rabbit IgG | FITC | 1:50 | eBioscience | 11-4839 |
| CCR7 | PE | 1:100 | BioLegend | 353204 |
| FOXP3 | PE | 1:20 | BD Pharmingen | 560046 |
| CD14 | PE | 1:100 | BioLegend | 301806 |
| CXCR3 | PE | 1:20 | BioLegend | 353706 |
| CD4 | PerCP-Cy5.5 | 1:100 | BioLegend | 300530 |
| 4-1BB | PerCP-Cy5.5 | 1:20 | BioLegend | 309814 |
| CD19 | PE-Cy7 | 1:20 | BioLegend | 302216 |
| CD45RA | PE-Cy7 | 1:100 | eBioscience | 25-0458 |
| CCR4 | PE-Cy7 | 1:100 | BioLegend | 359410 |
| CCR6 | PE-Cy7 | 1:20 | BioLegend | 353418 |
| CD25 | PE-Cy7 | 1:20 | BioLegend | 302612 |
| IL-2 | PE-Cy7 | 1:100 | BioLegend | 500326 |
| HLA-DQ | APC | 1:100 | eBioscience | 17-9881 |
| CD38 | APC | 1:20 | BioLegend | 356606 |
| CD127 | APC | 1:100 | BioLegend | 351316 |
| TNFa | APC | 1:100 | BioLegend | 502912 |
| CTLA4 | APC | 1:20 | BioLegend | 349908 |
| CD8 | APC-H7 | 1:100 | BD Pharmingen | 560273 |
| CD45RO | APC-Cy7 | 1:50 | BioLegend | 304228 |
| IFN-γ | APC-eFluor780 | 1:20 | eBioscience | 47-7319 |
| CD3 | V450 | 1:20 | BD Horizon | 560365 |
| CD8 | V450 | 1:50 | eBioscience | 48-0087 |
| HLA-DR | V500 | 1:20 | BD Horizon | 561224 |
| CD4 | BV650 | 1:100 | BioLegend | 317436 |
| CD16 | AF700 | 1:20 | BioLegend | 302026 |

**Supplementary Table 3. Immunophenotypic markers for innate and adaptive immune cells**. Innate and adaptive cells were defined using the cell surface markers defined by the Human Immunophenotype Project.

| ***Immunophenotype of Innate Cells*** | | | | | |
| --- | --- | --- | --- | --- | --- |
| **Cell** | **CD19** | **CD14** | **CD16** | **HLADR** | **HLADQ** |
| Classical Monocyte | - | + | - | + | + |
| Non-classical monocyte | - | + | + | + | + |
| B Cells | + | - | - | + | + |

|  | ***Immunophenotype of Adaptive Cells*** | | | | | | | | | | | | |
| --- | --- | --- | --- | --- | --- | --- | --- | --- | --- | --- | --- | --- | --- |
|  | **Cell** | **CD3** | **CD4** | **CD8** | **CCR7** | **CD45RA** | **CD45RO** | **CXCR3** | **CCR6** | **FOXP3** | **CD25** | **CCR4** | **CD127** |
|  |  |  |  |  |  |  |  |  |  |  |  |  |  |
|  | Tcm | + | + | + | + | - |  |  |  |  |  |  |  |
|  | Teff | + | + | + | - | + |  |  |  |  |  |  |  |
|  | Tem | + | + | + | - | - |  |  |  |  |  |  |  |
|  | Tnaive | + | + | + | + | + |  |  |  |  |  |  |  |
|  | Naïve Treg | + | + |  |  |  | - |  |  | + | + | + | - |
|  | Memory Treg | + | + |  |  |  | + |  |  | + | + | + | - |
|  | Th1 | + | + |  |  |  | + | + | - |  |  |  |  |
|  | Th2 | + | + |  |  |  | + | - | - |  |  |  |  |
|  | Th17 | + | + |  |  |  | + | - | + |  |  |  |  |
|  |  | | | | | | | | | | | | |
