# Supplementary material for: LRRK2 levels in immune cells are increased in Parkinson’s disease
Source: NPJ Parkinsons Dis. 2017 Mar 28;3:11. doi: 10.1038/s41531-017-0010-8 (PMC5459798; doi:10.1038/s41531-017-0010-8)
Supplement: Supplementary file 2 — Supplementary Figure Legends [file 41531_2017_10_MOESM2_ESM.docx]

**Supplementary Figures**

**Supplementary Figure S1. Detection of mouse LRRK2 protein by western blot and flow cytometry with the MJFF2 LRRK2 antibody is minimal.** PBMCs from LRRK2 knockout, wild-type C57BL/6J, mouse WT-LRRK2 BAC (Tg-OX), or mouse G2019S-LRRK2-overexpressing, and T cells, B cells, and monocytes isolated from human blood were obtained and processed for western blot analyses (a) or permeabilized and immunolabeled for flow cytometry (b) for detection of LRRK2 with the MJFF2 (Abcam c41-2) rabbit polyclonal antibody.

**Supplementary Figure S2. Protein levels of cell specific surface markers in B cells, monocytes, and T cells are not different between PD and HC subjects.** MFI of (a) CD19 in B cells (*p*=0.23, HC n=21, PD n=28), (b) CD14 in monocytes (*p*=0.94, HC n=31, PD n=36), and (c-d) 4-1BB in CD4^+^ (*p*=0.16, HC n=16, PD n=20) and CD8^+^ (*p*=0.21, HC n=15, PD n=20) T cells.

**Supplementary Figure S3. T cells frequencies are decreased in subjects with PD relative to HC subjects due to a decrease in CD4^+^ T cells.** (a) Frequencies as percentage of total peripheral blood mononuclear cells of monocytes (*p*=0.081, HC n= 31, PD n=36), B cells (*p*>0.05, HC n=22, PD n=28), and T cells (*p*=0.018, HC n=29, PD n=36), (b) a subset of monocytes (*p*>0.05, HC n= 18, PD n=29), (c) CD4^+^ helper subsets (Th1, *p*>0.05 HC n=27, PD n=34; Th2, *p*>0.05 HC n=27, PD n=34; Th17, *p*>0.05 HC n=27, PD n=34; Treg, *p*>0.05 HC n=26, PD n=31) were determined flow cytometry staining of total peripheral blood mononuclear cells, and (d) CD4^+^ (*p*=0.023, HC n=29, PD n=34) and CD8^+^ (CD8^+^, *p*>0.05 HC n=31, PD n=36) T cells subsets. (e) Frequencies of T cell effector subsets were determined as a percentage of CD4^+^ (CD4^+^ HC n=26, PD n=33, Tcm *p*>0.05; Naïve *p*>0.05; Tem *p*>0.05; Teff *p*>0.05) or CD8^+^ (CD8^+^ HC n=22, PD n=33, Tcm *p*>0.05; Naïve *p*>0.05; Tem *p*>0.05; Teff *p*>0.05) T cells, respectively. Means were plotted with standard error of the mean. Two-tailed Student’s t-test between HC and PD was used to test for significance. * *p* < 0.05.

**Supplementary Figure S4. Proliferation of T cells from PD and HC subjects is similar after 72 hrs but LRRK2 protein levels show different correlations with percentage of proliferated cells in PD versus HC subjects.** Percent of cells that proliferated after 72 hrs of stimulation with CD3/CD28 and IL-2 was calculated using CellTrace Violet and flow cytometry. (a) No differences between HC and PD seen for both CD4^+^ and CD8^+^ T cells. The percent of cells that divided at least three times also showed no differences between the two groups. Means were plotted with standard error of the mean. Two-tailed Student’s t-test between HC and PD was used to determine significance. *p* < 0.05. (b) Percentage of proliferating cells (CD4^+^ T cells: HC *R^2^*(11)=0.339, *p*=0.060, PD *R^2^*(7)=0.120, *p*=0.446; *F*(1, 14)=0.693, *p*=0.419) CD8^+^ T cells: HC *R^2^*(9)=0.038, *p*=0.614, PD *R^2^*(8)=0.293, *p*=0.166; *F*(1,13)=0.016, *p*=0.901) versus LRRK2 median fluorescence intensity (MFI) as measured by flow cytometry showed significant difference for CD4^+^ T cells between PD and HC. ANCOVA was performed to assess differences of slopes between HC and PD. Linear regression was used to assess individual correlations of slopes of HC and PD. Significance was set at *p* < 0.05.

**Supplementary Figure S5. Gating Strategy for Flow Cytometry Analysis.** Gating for (a) Live/dead and singlet cells, (b) monocytes, (c) Teff cells, (d) T helper cells, and (e) Treg cells. Cells were stained with fluorophore-conjugated antibodies to cell-surface or intracellular markers (See **Supplementary Table 2**) for flow cytometry and analyzed on a LSR-II flow cytometer (BD Bioscience) after standardization and compensation with Supra Rainbow Sphero beads and OneComp beads. Gates were placed based on staining with isotype control antibodies. Analysis was performed and plots were generated using FlowJo software.
