# Supplementary figures and images for: LRRK2 levels in immune cells are increased in Parkinson’s disease
Source: NPJ Parkinsons Dis. 2017 Mar 28;3:11. doi: 10.1038/s41531-017-0010-8 (PMC5459798; doi:10.1038/s41531-017-0010-8)

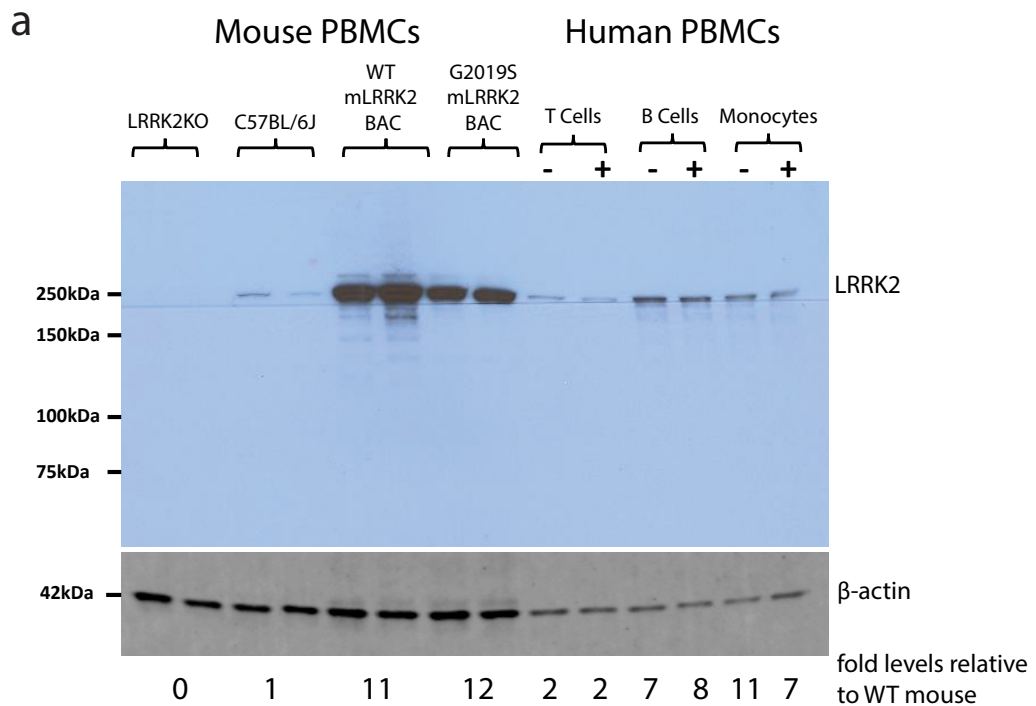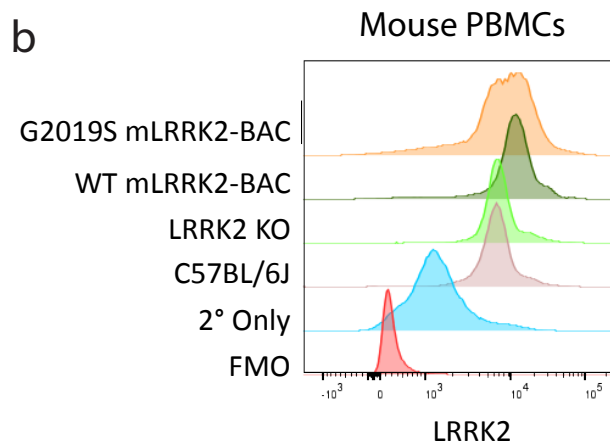

Supplementary Figure S1

Supplement: Supplementary file 3 — Supplementary Figure S1 [file 41531_2017_10_MOESM3_ESM.pdf]

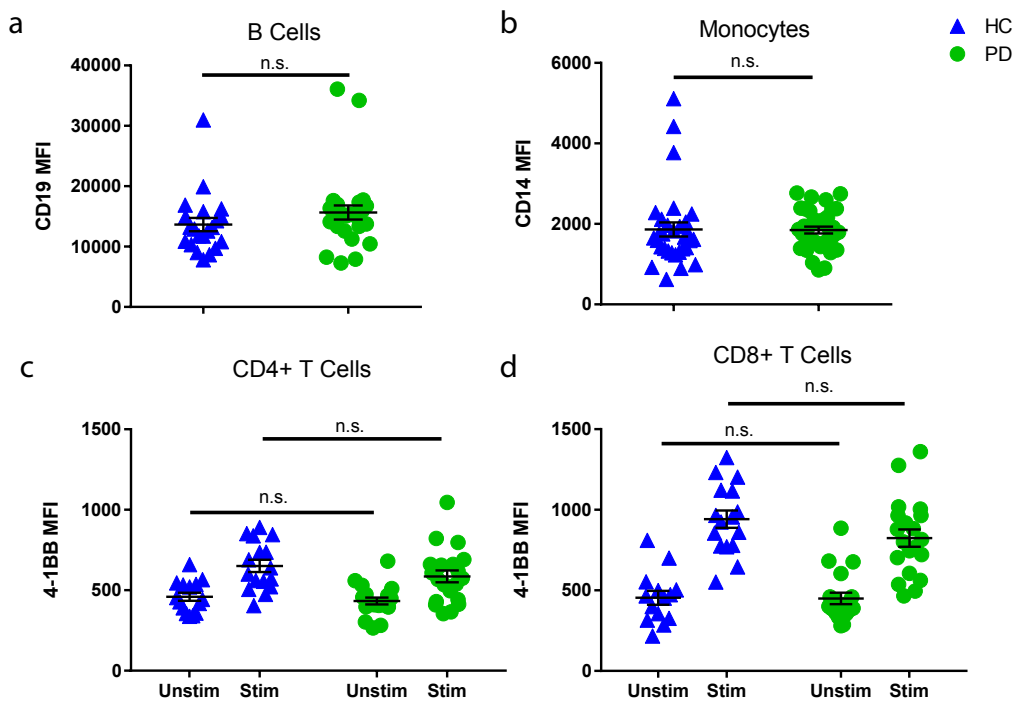

Supplementary Figure S2

Supplement: Supplementary file 4 — Supplementary Figure S2 [file 41531_2017_10_MOESM4_ESM.pdf]

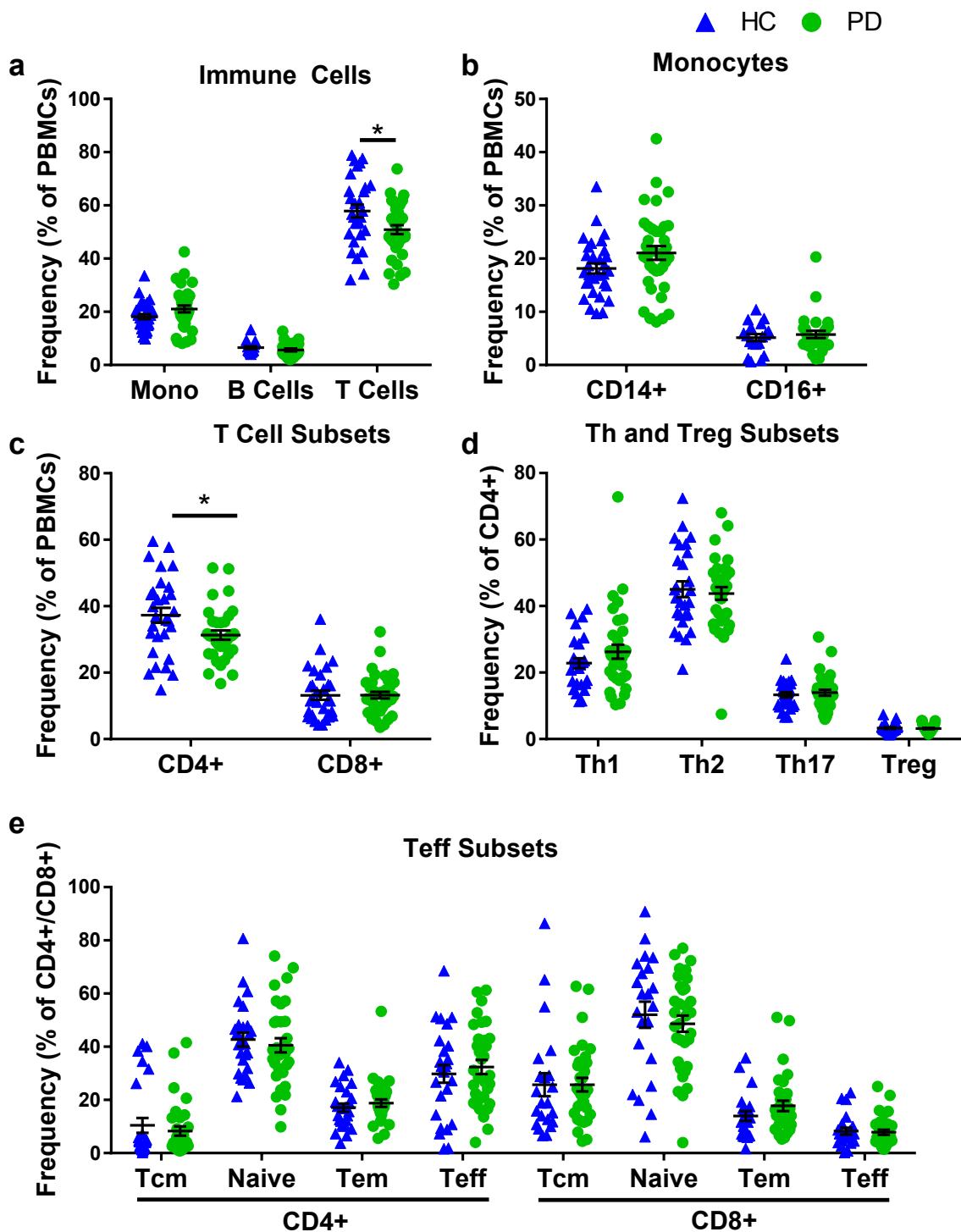

Supplementary Figure S3

Supplement: Supplementary file 5 — Supplementary Figure S3 [file 41531_2017_10_MOESM5_ESM.pdf]

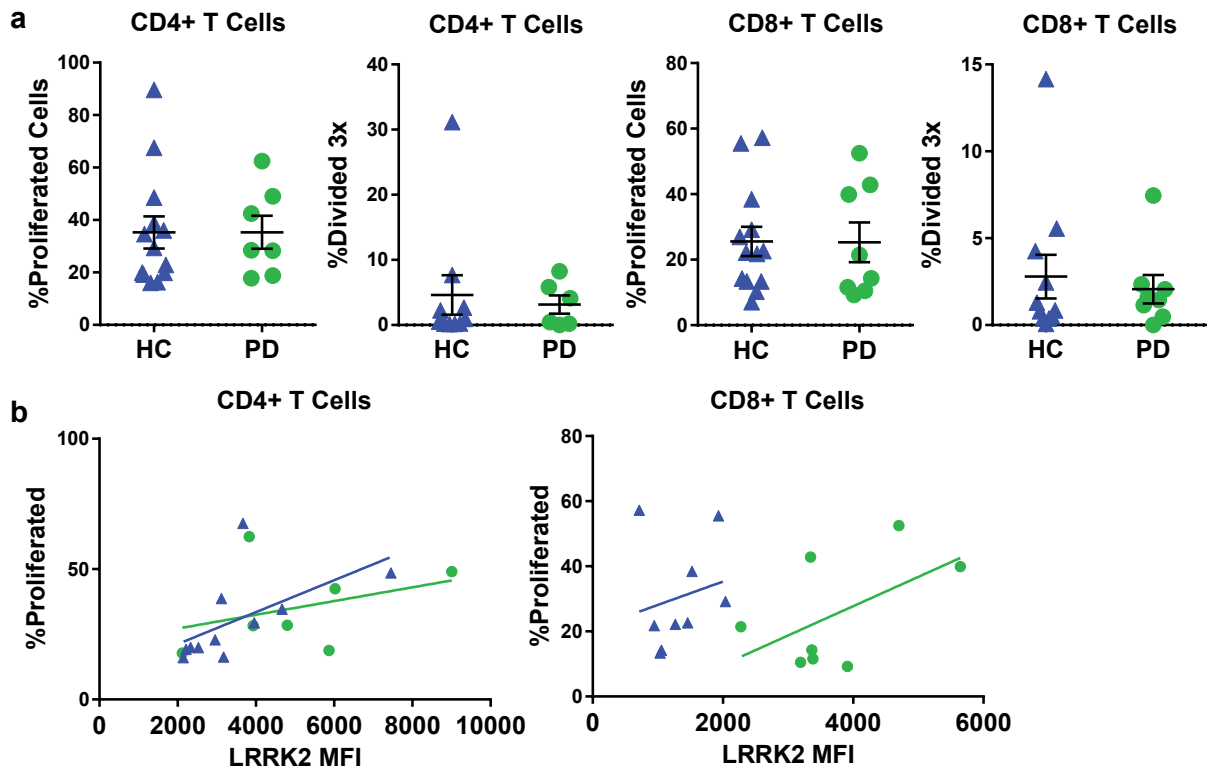

Supplementary Figure S

Supplement: Supplementary file 6 — Supplementary Figure S4 [file 41531_2017_10_MOESM6_ESM.pdf]

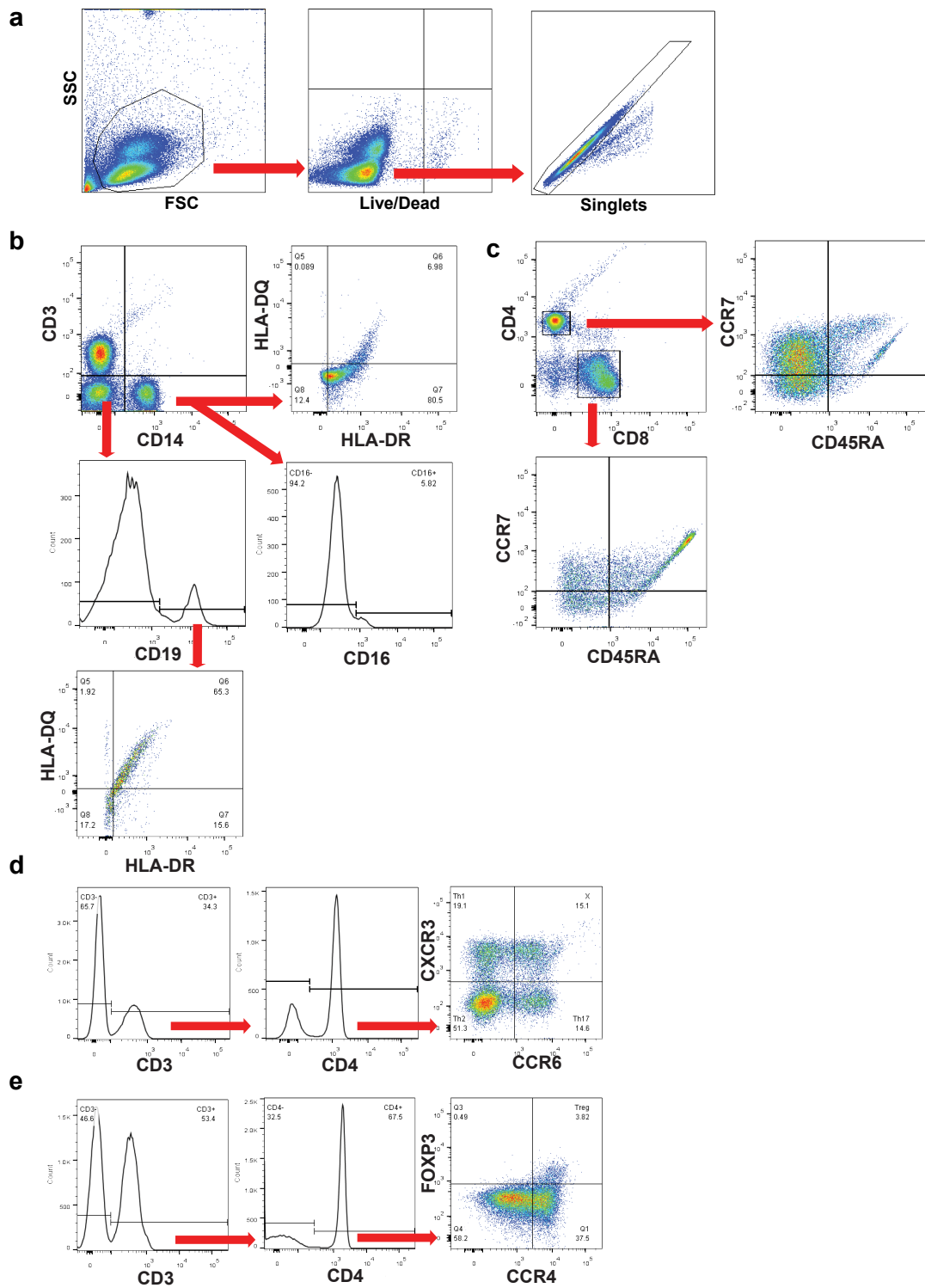

Supplementary Figure S5

Supplement: Supplementary file 7 — Supplementary Figure S5 [file 41531_2017_10_MOESM7_ESM.pdf]
